# Comparative Perioperative Inflammatory and Functional Outcomes in Single Versus Multiple Joint Replacements for Hemophilic Arthritis: A Pilot Study

**DOI:** 10.1111/os.70045

**Published:** 2025-05-08

**Authors:** Maoye Shen, Ping Qian, Wenxue Jiang, Shanyou Yuan, Gaorui Cai, Zhenzhong Zhou, Xiaona Wu, Jinghua Wang, Xianjia Ning, Lixia Song

**Affiliations:** ^1^ Department of Orthopedics Shenzhen Third People's Hospital and the Second Hospital Affiliated With the Southern University of Science and Technology Shenzhen Guangdong China; ^2^ Center of Clinical Epidemiology Shenzhen Third People's Hospital and the Second Hospital Affiliated With the Southern University of Science and Technology Shenzhen Guangdong China

**Keywords:** blood transfusion, hemophilic arthritis, joint replacement, perioperative inflammation, surgical outcomes

## Abstract

**Objective:**

Hemophilic arthritis is a progressive joint disease often requiring surgical intervention in advanced stages. However, comparative evidence on perioperative inflammatory and coagulation responses between single joint replacement (SJR) and multiple joint replacement (MJR) remains scarce. This study aimed to assess the differences in perioperative outcomes, including inflammatory responses, blood transfusion requirements, and functional recovery, to guide surgical decision‐making for hemophilic arthritis patients.

**Methods:**

This retrospective study included 29 male patients with moderate‐to‐severe hemophilic arthritis who underwent SJR (*n* = 12) or MJR (*n* = 17) at a single institution from October 2020 to October 2023. Data on inflammatory markers (CRP, ESR, IL‐6, WBC), hemoglobin levels, blood transfusion requirements, and joint mobility were collected for the immediate postoperative period (days 1–14). Trends in inflammatory markers were analyzed using average percent changes (APC), and differences in outcomes were evaluated using the Mann–Whitney *U* test for continuous variables and Fisher's exact test for categorical variables. Longitudinal changes were analyzed using mixed‐model repeated measures ANOVA with time points as fixed effects and subjects as random effects. Statistical significance was set at *p* < 0.05.

**Results:**

Postoperative CRP levels declined significantly in both groups, with APCs of −9.06% (95% CI: −15.63 to −1.98, *p* < 0.05) for the SJR group and −8.42% (95% CI: −16.18 to 0.06) for the MJR group. ESR showed a significant upward trend, with APCs of 10.82% (95% CI: 0.95–21.65, *p* < 0.05) in the SJR group and 17.54% (95% CI: 11.71–23.67, *p* < 0.05) in the MJR group. Blood transfusion requirements were comparable, with median transfusion volumes of 0.00 units (IQR: 3.50) for SJR and 0.00 units (IQR: 3.75) for MJR (*p* = 0.761). Notably, joint mobility scores were significantly better in the MJR group (mean: 31.88, SD: 19.31) compared to the SJR group (mean: 18.33, SD: 10.39; *p* = 0.030). Despite the larger surgical scope of MJR, no significant differences in infection or bleeding risks (SJR:median transfusion = 0.00 units, IQR: 3.50; MJR:median transfusion, 0.00 units, IQR: 3.75. *p* = 0.761) were observed between the groups.

**Conclusion:**

This study demonstrates that MJR offers superior functional recovery compared to SJR, without increasing the risks of infection, bleeding, or transfusion. These findings support MJR as a safe and effective surgical option for hemophilic arthritis patients when appropriate perioperative management protocols are implemented. Future studies with larger sample sizes and long‐term follow‐up are needed to validate these results and explore extended outcomes.

## Graphical abstract

This study reveals that, compared to single‐joint replacement, multi‐joint replacement enhances joint mobility in hemophilia patients without additional postoperative infection or transfusion risks.

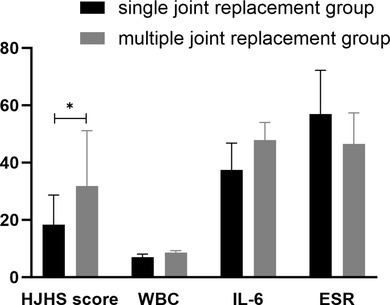

## 1 Introduction

Hemophilia is a genetically inherited X‐linked bleeding disorder that primarily manifests as Hemophilia A, caused by factor VIII deficiency, and Hemophilia B, resulting from factor IX deficiency [1]. A major complication of this condition is hemophilic arthropathy, which arises from recurrent spontaneous intra‐articular hemorrhages, progressively damaging joint structures. Over time, this leads to persistent joint pain, significant limb deformities, and severe functional impairments [2]. The knees, ankles, and elbows are the most commonly affected joints, with multiple joint involvement frequently observed [2, 3]. Advanced hemophilic arthropathy often necessitates joint replacement surgery, a procedure that significantly enhances patients' quality of life but simultaneously imposes substantial healthcare burdens [2, 3].

Joint replacement surgery has become the cornerstone treatment for advanced hemophilic arthritis, demonstrating improved clinical outcomes in recent years [4, 5]. Progress in surgical techniques and perioperative management strategies has further optimized patient care, addressing postoperative hematologic and inflammatory responses [6]. However, while there has been extensive research on surgical outcomes in hemophilic patients, studies directly comparing Single Joint Replacement (SJR, defined as the replacement of one major joint in a single surgical session) and concurrent Multiple Joint Replacements (MJR, defined as the replacement of two or more joints in a single surgical session) in this unique population remain scarce.

A critical gap in the literature is the limited understanding of the differential inflammatory and coagulative responses associated with SJR versus MJR in hemophilic patients. Previous studies have primarily focused on broad surgical outcomes, often overlooking how the extent of surgical intervention influences these physiological responses. This is particularly relevant given the heightened vulnerability of hemophilic patients to infections and comorbid conditions due to impaired immunity and frequent perioperative transfusions [6, 7, 8]. Furthermore, variability in perioperative management, including the administration of coagulation factors and blood transfusions, adds another layer of complexity to interpreting outcomes [9]. Robust data comparing SJR and MJR are needed to refine treatment protocols and support evidence‐based clinical decision‐making.

This study hypothesizes that SJR will elicit inflammatory and hematologic responses comparable to those observed in multiple joint replacements due to focused perioperative management and a more localized surgical trauma. To test this hypothesis, the objective of this study is to evaluate and compare postoperative hematologic and inflammatory responses in hemophilic arthritis patients undergoing SJR versus MJR. Specifically, this study will analyze baseline preoperative characteristics and assess trends in key laboratory indicators during the immediate postoperative period, spanning 1–14 days.

## 2 Materials and Methods

### 2.1 Study Design

This retrospective study aimed to evaluate the comparative outcomes of single versus MJR surgeries in patients with hemophilic arthritis, with a primary focus on postoperative anemia and infection rates.

### 2.2 Patient Selection

Data were obtained from hemophilic patients admitted to the Department of Orthopedics at Shenzhen Third People's Hospital for hip and knee joint replacement surgeries between October 2020 and October 2023. All patients included were male, had congenital moderate‐to‐severe hemophilia, and met the following eligibility criteria: a definitive diagnosis of hemophilic arthritis, a documented history of joint pain and dysfunction, and imaging evidence confirming end‐stage joint arthropathy. Patients were excluded if they had non‐hemophilic arthritis, peripheral neuropathy, other neurological dysfunctions, active infections elsewhere, or incomplete preoperative or postoperative medical records.

Patient demographics (e.g., age, body mass index (BMI)), medical history (type and severity of hemophilia, prior treatments), and surgical details (site, timing, incision type, and number of joints replaced) were extracted from hospital records. The study complied with the Declaration of Helsinki and was approved by the Ethics Committee of Shenzhen Third People's Hospital. Written informed consent was obtained from all participants.

### 2.3 Data Collection and Validation

Data for this study were retrospectively collected from the hospital's electronic medical records, surgical logs, and laboratory reports. A standardized data collection form was developed specifically for this study to minimize information bias. This form included predefined fields for patient demographics, medical history, surgical details, and laboratory outcomes.

Data extraction was performed by two independent data administrators trained in data abstraction techniques. To ensure accuracy, all data were cross‐checked against multiple sources. For instance, demographic data were validated by comparing electronic medical records with surgical logs, while laboratory values were verified using laboratory reports and patient charts. Any discrepancies were resolved through consultation with a third independent reviewer.

### 2.4 Surgical Procedures

All surgical protocols adhered to standardized treatment guidelines for hemophilic patients. Preoperative optimization of coagulation factor levels, intraoperative bleeding control (e.g., tranexamic acid administration), and postoperative rehabilitation protocols were implemented uniformly. Decisions regarding single or multiple joint replacements were made by a multidisciplinary team, considering clinical necessity, patient preference, and overall health status.

Tranexamic acid was used to minimize intraoperative blood loss and reduce coagulation factor requirements. It was administered via intravenous infusion (15 mg/kg or 1 g prior to skin incision or 30 min before surgery), topical application (1–3 g retention perfusion), or oral dosing (1 g one to three times within 2 h before surgery and once again within 24 h post‐surgery). Tranexamic acid was avoided in patients with hemophilia B treated with prothrombin complex, recent thromboembolism, or a history of thromboembolic events. All surgeries were performed by the same team of experienced orthopedic surgeons.

Prophylactic second‐generation cephalosporins were administered 30 min before surgery and continued for 48 h postoperatively. Negative pressure drainage tubes were removed within 48 h, and oral anticoagulants were initiated 12 h after surgery, continuing for 35 days. Dressings were changed regularly, and stitches were removed after 2 weeks. Patients received standardized rehabilitation exercises under the guidance of the same medical team.

Hip replacements utilized a posterolateral approach, while knee replacements employed a median anterior incision.

### 2.5 Outcome Measures

Primary outcomes included postoperative hemoglobin levels and inflammation markers, namely white blood cell count (WBC), C‐reactive protein (CRP), erythrocyte sedimentation rate (ESR), and procalcitonin levels. These parameters were measured preoperatively, immediately postoperatively, and on postoperative days 1, 7, and 14. Secondary outcomes included pain scores, range of motion, functional status, and complications such as bleeding, transfusion requirements, or thromboembolic events. The pain score was assessed using the pain subscale of the Hemophilic Joint Health Score (HJHS), and range of motion and functional status were evaluated using the total score of the HJHS.

### 2.6 Definitions

Hemophilic arthritis was diagnosed based on family history, clinical presentation, and laboratory findings. Pain was assessed using a numeric rating scale (0–10). Functional status and joint mobility were evaluated using the Hemophilia Joint Health Score and the Gait Test Score.

Complications included postoperative bleeding, defined as a hemoglobin drop ≥ 2 g/dL within 24 h or requiring ≥ 2 units of packed red blood cells. Thromboembolic events referred to abnormal blood clot formation in deep veins impairing venous return. Antibiotic use was recorded only for postoperative administration, excluding preoperative prophylaxis. Signs of infection, such as fever, wound redness, swelling, or increased pain, prompted antibiotic treatment.

The APC was calculated to summarize trends in hematological and inflammatory markers over predefined time points (days 1–14). Positive APC values indicated an upward trend, while negative values denoted a decline.

### 2.7 Statistical Analysis

Continuous variables with normal distribution were expressed as mean ± standard deviation, while non‐normally distributed variables were presented as median and interquartile ranges. Categorical variables were summarized as counts and percentages. Group comparisons were conducted using the Mann–Whitney *U* test for continuous variables and Fisher's exact test for categorical variables, suitable for small‐sample studies.

Longitudinal changes in hematologic and inflammatory markers were analyzed using mixed‐model repeated measures ANOVA, with time points as fixed effects and subjects as random effects, employing the Restricted Maximum Likelihood (REML) method. Statistical significance was set at *p* < 0.05. All analyses were conducted using SPSS 25.0 (IBM Corporation, Armonk, NY, USA), and APC calculations were performed with Joinpoint 5.3.0 software (National Cancer Institute, Bethesda, MD, USA).

## 3 Results

### 3.1 Patient Demographics and Preoperative Characteristics

A total of 29 patients with hemophilic arthritis were included in the study. Among them, 12 patients (41.4%) underwent single joint replacement, with 8 cases being knee replacements. Seventeen patients (58.6%) underwent multiple joint replacements, including 1 case of bilateral hip replacement, 9 cases of hip and knee replacements, and 7 cases of bilateral knee replacements. The joint replacement sites of the two groups were comparable (both *p* > 0.05). The two groups were comparable in terms of age, with a mean of 33.25 years (SD = 9.70) for the single joint replacement group and 35.12 years (SD = 7.33) for the multiple joint replacement group (*p* = 0.558). However, surgery duration was significantly longer in the multiple joint replacement group (median: 282.00 min, IQR = 95.00) compared to the single joint replacement group (median: 112.50 min, IQR = 86.75; *p* = 0.002).

Preoperative laboratory indicators, including hemoglobin (HGB), ESR, WBC, CRP, interleukin‐6 (IL‐6), procalcitonin (PCT), D‐dimer, activated partial thromboplastin time (APTT), prothrombin time (PT), fibrinogen, and clotting factor levels, showed no significant differences between the groups, indicating a consistent baseline (Table 1).

**TABLE 1:** Preoperative information.

Laboratory indicators	Single joint replacement	Multiple joint replacement	*p*
Age, means (SD), years	33.25 (9.70)	35.12 (7.33)	0.558
Surgery times, median (IQR), minutes	112.50 (86.75)	282.00 (95.00)	0.002
Surgery site, *n* (%):			1.000
Hip	4 (33.3)	10 (58.8)	0.264
Knee	8 (66.7)	16 (94.1)	0.130
HGB, median (IQR), g/L	150.50 (26.25)	143.00 (13.00)	0.626
ESR, median (IQR), mm/h	16.00 (11.50)	13.00 (21.50)	0.610
WBC, median (IQR), 10^9/L	4.95 (2.55)	5.82 (2.30)	0.330
CRP, median (IQR), mg/L	1.81 (3.95)	1.94 (3.00)	1.000
IL‐6, median (IQR), Pg/ml	2.20 (2.83)	4.16 (4.66)	0.265
PCT, median (IQR), ng/mL	0.15 (0.22)	0.04 (0.08)	0.089
D‐Dimmer, median (IQR), ng/mL	0.22 (0.33)	0.22 (0.28)	0.814
APTT, means (SD), *s*	67.90 (23.20)	80.58 (27.55)	0.403
PT, median (IQR), *s*	12.83 (1.22)	13.15 (1.05)	0.218
Fibrinogen, median (IQR), g/L	2.76 (0.53)	2.38 (1.49)	0.210
Clotting factors test, median (IQR)	1.00 (1.00)	1.00 (1.00)	0.431

Abbreviations: APTT, Activated partial thromboplastin time; CRP, C‐reactive protein; ESR, erythrocyte sedimentation rate; HGB, hemoglobin; IL‐6, interleukin‐6; PCT, procalcitonin; PT, Prothrombin time; WBC, white blood cell count.

### 3.2 Postoperative Hematologic and Inflammatory Response

Postoperative trends in hematologic and inflammatory markers are summarized in Table 2. In the SJR replacement group, ESR showed a significant upward trend, with an APC of 10.82% (95% CI: 0.95–21.65; *p* < 0.05). Conversely, CRP, IL‐6, and PCT levels decreased significantly, with APCs of −9.06% (95% CI: −15.63 to −1.98; *p* < 0.05), −17.36% (95% CI: −25.79 to −7.98; *p* < 0.05), and − 10.10% (95% CI: −14.82 to −5.13; *p* < 0.05), respectively.

**TABLE 2:** Trends in hemoglobin and inflammatory indicators.

Laboratory indicators	Postoperative days								
	1	2	3	4	5	6	7	14	APC
Single joint replacement									
HGB	116.00 (11.37)^a^	98.78 (16.47)^a^	97.30 (13.91)^a^	93.22 (13.41)^a^	97.00 (37.20)	89.17 (11.99)^a^	100.71 (10.37)^a^	105.86 (11.13)^a^	−0.17 (−2.24, 1.94)
ESR	9.00 (13.00)	39.61 (22.54)^a^	36.48 (27.65)^a^	74.13 (22.86)^a^	77.00 (13.50)^a^	74.00 (26.99)^a^	68.00 (46.00)^a^	68.64 (27.66)^a^	10.82 (0.95, 21.65)*
WBC	10.91 (1.99)^a^	8.50 (2.71)^a^	7.62 (2.55)^a^	7.85 (2.49)^a^	7.97 (2.93)^a^	5.80 (3.04)^a^	6.44 (2.08)^a^	7.11 (1.46)^a^	−2.65 (−6.29, 1.14)
CRP	72.27 (44.93)^a^	125.46 (16.53)^a^	108.28 (42.44)^a^	80.55 (43.45)^a^	93.96 (81.37)	53.16 (27.32)^a^	35.50 (24.50)^a^	33.53 (27.63)^a^	−9.06 (−15.63, −1.98)*
IL‐6	65.35 (51.94)^a^	43.00 (83.00)	65.39 (23.91)^a^	23.00 (27.40)	18.60 (12.04)	16.29 (8.52)^a^	9.08 (3.09)^a^	5.60 (8.70)	−17.36 (−25.79, −7.98)*
PCT	0.36 (0.21)^a^	0.23 (0.56)	0.26 (0.18)^a^	0.13 (0.42)	0.24 (0.14)^a^	0.19 (0.14)^a^	0.14 (0.18)	0.08 (0.15)	−10.10 (−14.82, −5.13)*
D‐Dimmer	3.77 (6.71)	2.00 (1.43)	2.33 (1.24)^a^	2.45 (1.54)^a^	2.63 (2.25)^a^	0.90 (3.04)	1.30 (5.60)	4.11 (8.06)	−4.85 (−11.38, 2.16)
Multiple joint replacement									
HGB	111.12 (14.59)^a^	90.13 (17.70)^a^	78.64 (18.00)^a^	85.40 (21.21)^a^	82.00 (12.44)^a^	84.50 (11.41)^a^	92.29 (13.77)^a^	101.30 (13.42)^a^	−0.47 (−2.11, 1.19)
ESR	3.50 (5.75)	6.00 (67.00)	43.00 (30.50)	49.67 (18.93)^a^	60.50 (23.75)^a^	63.67 (26.34)^a^	70.50 (38.25)^a^	70.36 (25.67)^a^	17.54 (11.71, 23.67)*
WBC	13.64 (3.83)^a^	10.94 (4.22)^a^	9.12 (3.74)^a^	8.46 (3.94)^a^	7.56 (3.11)^a^	6.74 (2.38)^a^	8.58 (2.86)^a^	7.26 (2.11)^a^	−4.45 (−7.43, −1.36)*
CRP	61.74 (31.59)^a^	81.65 (42.96)^a^	121.90 (72.26)^a^	90.76 (55.44)^a^	104.23 (63.45)^a^	97.09 (71.29)^a^	48.21 (66.44)	17.91 (13.75)^a^	−8.42 (−16.18, 0.06)
IL‐6	60.35 (60.73)	57.20 (37.80)	68.00 (39.70)	30.00 (33.50)	20.00 (12.65)	18.60 (8.20)	10.30 (13.44)	5.20 (10.75)	−17.66 (−24.22, −10.53)*
PCT	0.19 (0.67)	0.20 (0.49)	0.27 (0.36)	0.31 (0.22)^a^	0.22 (0.14)^a^	0.15 (0.29)	0.12 (0.22)	0.12 (0.25)	−3.60 (−9.71, 2.93)
D‐Dimmer	5.00 (4.67)	2.30 (1.97)^a^	3.09 (1.22)^a^	1.93 (1.48)^a^	2.96 (2.47)^a^	2.30 (4.64)	5.32 (3.04)^a^	3.09 (355)	0.44 (−8.12, 9.84)

Abbreviations: CRP, C‐reactive protein; ESR, erythrocyte sedimentation rate; HGB, hemoglobin; IL‐6, interleukin‐6; PCT, procalcitonin; WBC, white blood cell count.

^a^ Indicates that the data conforms to the normal distribution, and the absence of “a” indicates that the data does not conform to the non‐normal distribution.

* *p* < 0.05.

**FIGURE 1:**
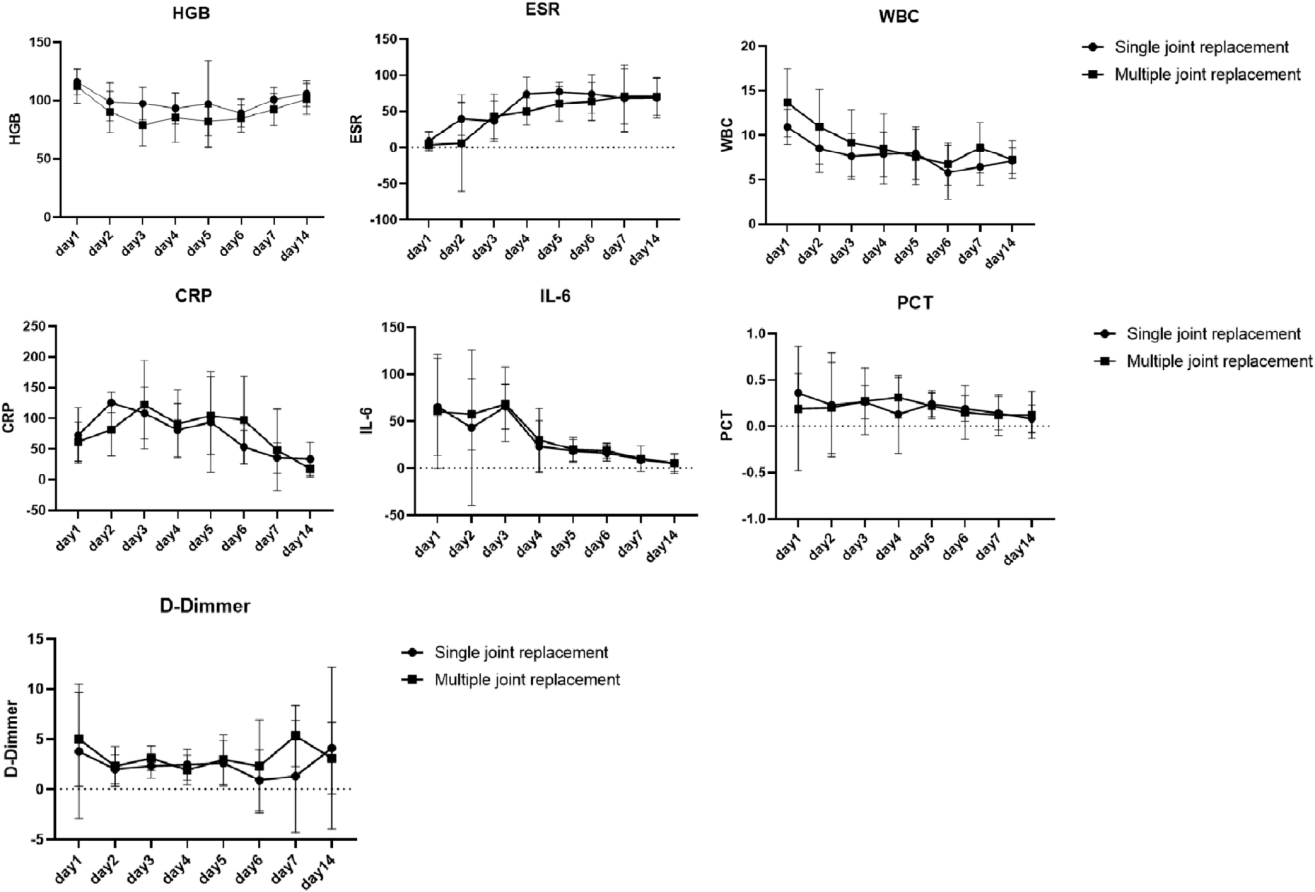
Postoperative hematologic and inflammatory response between single joint replacement group and multiple joint replacement group.

In the MJR group, ESR also exhibited a significant upward trend (APC: 17.54%, 95% CI: 11.71–23.67; *p* < 0.05). Meanwhile, WBC and IL‐6 levels decreased significantly, with daily APCs of −4.45% (95% CI: −7.43 to −1.36; *p* < 0.05) and −17.66% (95% CI: −24.22 to −10.53; *p* < 0.05), respectively (Figure 1).

### 3.3 Comparative Analysis of Laboratory Indicator Trends

The trends in laboratory markers over the 14‐day postoperative period were compared between the two groups (Table 3). No significant differences were observed in HGB (Difference = 7.69, 95% CI: −3.63 to 19.02, *p* = 0.156), ESR (Difference = 10.38, 95% CI: −228.96 to 249.71, *p* = 0.679), WBC (Difference = −1.60, 95% CI: −4.65 to 1.45, *p* = 0.260), IL‐6 (Difference = −10.44, 95% CI: −33.99 to 13.11, *p* = 0.364), PCT (Difference = −0.05, 95% CI: −0.25 to 0.14, *p* = 0.094), or D‐dimer (Difference = 0.90, 95% CI: −1.13 to 2.94, *p* = 0.370).

**TABLE 3:** Difference analysis of trends in laboratory indicators between two groups of subjects from 1 to 14 days.

Laboratory indicators	Single joint replacement	Multiple joint replacement	Difference (95% CI)	*p*
HGB	96.63 (4.39)	88.93 (2.20)	7.69 (−3.63, 19.02)	0.156
ESR	56.88 (15.38)	46.50 (10.88)	10.38 (−228.96, 249.71)	0.679
WBC	6.96 (1.11)	8.56 (0.72)	−1.60 (−4.65, 1.45)	0.260
CRP	—	—	—	—
IL‐6	37.43 (9.38)	47.87 (6.14)	−10.44 (−33.99, 13.11)	0.364
PCT	0.28 (0.07)	0.34 (0.06)	−0.05 (−0.25, 0.14)	0.094
D‐Dimmer	4.43 (0.79)	3.53 (0.60)	0.90 (−1.13, 2.94)	0.370

Abbreviations: CRP, C‐reactive protein; ESR, erythrocyte sedimentation rate; HGB, hemoglobin; IL‐6, interleukin‐6; PCT, procalcitonin; WBC, white blood cell count.

### 3.4 Secondary Outcomes and Complications

Secondary outcomes, including postoperative pain scores, range of motion, functional status, and complications, are detailed in Table 4. The postoperative pain scores were evaluated using the pain subscale of the Hemophilic Joint Health Score (HJHS), and the results showed no significant difference between the single joint replacement (SJR) group and the multiple joint replacement (MJR) group (median for SJR: 2.00, interquartile range [IQR]: 3.75; median for MJR: 2.00, IQR: 4.00, *p* = 0.873). Range of motion and functional status were assessed using the total score of the HJHS, and the MJR group significantly outperformed the SJR group in these metrics (mean for SJR: 18.33, standard deviation [SD]: 10.39; mean for MJR: 31.88, SD: 19.31; *p* = 0.030). Regarding blood transfusion requirements, there was no significant difference between the two groups (median for SJR: 0.00, IQR: 3.50; median for MJR: 0.00, IQR: 3.75, *p* = 0.761). Similarly, there was no significant difference in the use of antibiotics between the groups (7 cases, 58.3% in the SJR group; 10 cases, 58.8% in the MJR group; *p* = 0.637).

**TABLE 4:** Secondary outcomes and complications between two groups.

Subjects	Single joint replacement	Multiple joint replacement	*p*
Postoperative pain scores, median (IQR)	2.00 (3.75)	2.00 (4.00)	0.873
Range of motion and functional status, means (SD)	18.33 (10.39)	31.88 (19.31)	0.030
The amount of blood transfused, median (IQR)	0.00 (3.50)	0.00 (3.75)	0.761
Use of antibiotics, *n* (%)	7 (58.3)	10 (58.8)	0.637

During the postoperative monitoring period from day 1 to day 14, no cases of thromboembolic events or excessive bleeding were observed. Additionally, there were no records of hemoglobin levels dropping by ≥ 2 g/dL within 24 h or requiring a transfusion of ≥ 2 units of packed red blood cells, nor were there any occurrences of abnormal blood clots in the deep veins that could impair venous return. No such complications were reported in either group throughout the monitoring period.

### 3.5 Molecular‐Level Changes on the First Postoperative Day

On the first postoperative day, HGB levels significantly decreased in both groups compared to preoperative values (*p* < 0.05). Inflammatory markers, including WBC, CRP, IL‐6, PCT, and D‐dimer, showed significant increases in both groups (all *p* < 0.05). Interestingly, ESR decreased significantly in the MJR group (*p* = 0.014) but not in the SJR group (Table 5).

**TABLE 5:** Molecular level changes during preoperative and first postoperative day changes.

Laboratory indicators	Preoperative	First postoperative day	*p*
Single joint replacement			
HGB	150.50 (26.25)	116.00 (11.37)	0.002
ESR	16.00 (11.50)	9.00 (13.00)	0.189
WBC	4.95 (2.55)	10.91 (1.99)	< 0.001
CRP	1.81 (3.95)	72.27 (44.93)	0.002
IL‐6	2.20 (2.83)	65.35 (51.94)	0.003
PCT	0.15 (0.22)	0.36 (0.21)	0.006
D‐Dimmer	0.22 (0.33)	3.77 (6.71)	< 0.001
Multiple joint replacement			
HGB	143.00 (13.00)	111.12 (14.59)	< 0.001
ESR	13.00 (21.50)	3.50 (5.75)	0.014
WBC	5.82 (2.30)	13.64 (3.83)	< 0.001
CRP	1.94 (3.00)	61.74 (31.59)	< 0.001
IL‐6	4.16 (4.66)	60.35 (60.73)	< 0.001
PCT	0.04 (0.08)	0.19 (0.67)	< 0.001
D‐Dimmer	0.22 (0.28)	5.00 (4.67)	< 0.001

Abbreviations: CRP, C‐reactive protein; ESR, erythrocyte sedimentation rate; HGB, hemoglobin; IL‐6, interleukin‐6; PCT, procalcitonin; WBC, white blood cell count.

## 4 Discussion

### 4.1 Results Interpretation

This study aimed to evaluate and compare perioperative inflammatory and coagulation responses in patients with hemophilic arthritis undergoing SJR versus MJR. Secondary outcomes included pain scores, range of motion, functional status, and complications such as bleeding, transfusion requirements, or thromboembolic events. The key findings of this study indicate that there were no significant differences in the risk of infection or bleeding between patients undergoing SJR and those undergoing MJR. However, joint mobility outcomes were significantly better in the MJR group. These findings carry important clinical implications, particularly for the perioperative management of hemophilic patients requiring joint replacement surgery.

### 4.2 Inflammatory Responses

The relationship between single and MJR surgeries and postoperative inflammatory responses has been well documented. Prior studies have explored how varying surgical approaches influence inflammatory markers, providing a basis for comparing our findings. For instance, one study observed that hemophilic patients undergoing joint replacements commonly exhibit elevated ESR and CRP levels postoperatively, indicative of an acute inflammatory response to surgical trauma [10]. Similarly, joint replacement surgeries, regardless of the number of joints replaced, have been shown to trigger significant inflammatory responses, with CRP levels serving as a reliable marker for monitoring postoperative inflammation [11]. Another study underscored the importance of tracking inflammatory markers in hemophilic patients undergoing orthopedic surgeries to prevent complications such as infections and prolonged recovery times [12].

In comparison to these findings, our study revealed that the inflammatory responses observed after SJR were similar to those following MJR. This contrasts with the general expectation that more extensive surgeries, like MJR, would elicit stronger inflammatory responses. One possible explanation is that, while MJR is associated with a broader range of surgical trauma, the localized and concentrated trauma of SJR may provoke a more intense inflammatory reaction. Previous studies have suggested that localized trauma can trigger a stronger inflammatory response [10, 11, 12]. These findings provide a valuable foundation for refining surgical treatment strategies in patients with hemophilic arthritis.

### 4.3 Blood Transfusion Requirements

The need for blood transfusions in joint replacement surgeries has also been extensively reported in the literature. Several studies have highlighted the association between extensive surgical procedures and increased transfusion requirements, particularly in patients with underlying conditions like hemophilia. For instance, one study found that hemophilic patients undergoing total knee arthroplasty required significantly more blood transfusions than non‐hemophilic patients due to their inherent bleeding tendencies and the extensive nature of the surgery [13]. Similarly, Shurkhina et al. (2016) demonstrated that hemophilic patients undergoing joint replacement experienced greater intraoperative blood loss, necessitating higher transfusion volumes to achieve perioperative hemostasis [14]. Hu et al. (2018) also identified several factors influencing blood loss in total knee arthroplasty, emphasizing the need for tailored blood management strategies in hemophilic patients [15].

Contrary to these findings, our study observed no significant difference in postoperative blood transfusion requirements between the SJR and MJR groups. This challenges the expectation that MJR, being a more extensive procedure, would necessitate higher transfusion volumes. A possible explanation is that the focused surgical trauma in SJR may result in transfusion requirements comparable to the more extensive injury of MJR. This finding underscores that MJR in hemophilic patients does not inherently pose a higher risk of blood transfusion, offering reassurance for its broader application in this population.

### 4.4 Joint Mobility Assessment

Assessing joint mobility in hemophilic patients is a critical component of postoperative evaluation, with tools like the Hemophilia Joint Health Status Score and Gait Test score serving as reliable first‐line assessment methods [16, 17]. Previous studies have shown no significant differences in joint function between unilateral and bilateral joint replacements in hemophilic arthritis patients. For instance, one study on total knee arthroplasty reported similar joint function outcomes regardless of whether the surgery was unilateral or multilateral [18]. Another study found no significant differences in joint mobility or quality of life between single and multiple joint replacements [19].

In contrast to these findings, our study demonstrated that the MJR group had better postoperative range of motion compared to the SJR group. This may be attributed to the localized and intense inflammatory response in SJR, which could negatively impact range of motion. These findings highlight the potential benefits of MJR in improving functional outcomes in hemophilic arthritis patients, challenging existing assumptions and emphasizing the need for further investigation.

### 4.5 Strengths and Limitations

#### 4.5.1 Our Research Has Several Strengths

First, our study provides a unique comparative analysis between single joint replacement (SJR) and multiple joint replacement (MJR) in patients with hemophilic arthritis, which is a significant contribution to the field given the scarcity of such comparisons in existing literature. This analysis offers valuable insights into the relative benefits and risks of these surgical approaches.

Second, our comprehensive data collection, which includes a wide range of outcomes such as inflammatory markers, blood transfusion requirements, and functional outcomes, allows for a thorough analysis of the perioperative period. This depth of data is crucial for understanding the complex physiological responses to surgery in this patient population.

Third, the findings of our study have direct implications for clinical decision‐making, potentially influencing surgical strategies and patient outcomes, thereby highlighting the study's clinical relevance.

Last, the rigorous methodology employed in our study, including the use of robust statistical methods to analyze the data, ensures the reliability and validity of our results. This methodological rigor is essential for drawing accurate conclusions and for the study's contribution to evidence‐based clinical practice.

This study has several notable limitations that warrant consideration.

First, the relatively small sample size (29 patients) limits the generalizability of our findings and reduces the statistical power to detect subtle differences between the single and MJR groups. This small cohort may lead to overestimation or underestimation of the true effects of single versus multiple joint replacements on postoperative inflammatory responses and transfusion needs. The small sample size also precludes us from making between‐group comparisons of hip versus knee joints individually, and while we performed an inter‐group comparison of joint replacement scores between the two groups preoperatively and confirmed the comparability of joint scores between the two groups, this may still increase participant heterogeneity to some extent and have a potential impact on our findings, potentially introducing confounders and bias when comparing outcomes of postoperative blood loss or transfusion. Larger experiments should be conducted in the future, and subgroup analyses of hip and knee arthroplasties separately should be performed to validate our conclusions. Second, the retrospective study design introduces the potential for recall bias and limits our ability to control for confounding variables comprehensively. The accuracy of the data relied on the completeness and reliability of existing medical records, which may introduce inconsistencies. A prospective study design would allow for more precise data collection and the ability to adjust for confounding factors effectively.

Third, the study was conducted at a single institution, which may limit the external validity and broader applicability of our results. Multicenter studies involving diverse populations would enhance the generalizability of findings.

Fourth, patient information collected for this study was not exhaustive. Variables such as past medical history, medication use, and lifestyle factors were not included, potentially leaving unaccounted influences on perioperative outcomes. Incorporating these variables into future analyses could provide a more comprehensive understanding of patient‐related factors impacting surgical outcomes.

Fifth, although data on surgical approach and duration were collected, the small sample size limited meaningful stratified analyses based on these variables. As a result, we were unable to fully evaluate whether factors such as surgical approach, duration, or specific joint replacement sites (e.g., hip versus knee) influenced outcomes like blood loss, transfusion requirements, or postoperative inflammatory responses (e.g., ESR, CRP). Expanding the sample size in future research would enable robust subgroup analyses to assess these factors' impacts more comprehensively.

Another limitation involves the influence of local trauma and surgical stress on inflammatory markers such as CRP. While the study aimed to assess the perioperative inflammatory response in hemophilic arthritis patients, localized surgical effects, including tissue damage and stress, may have contributed to elevated inflammatory markers irrespective of the number of joints replaced. This potential confounder complicates the interpretation of our findings, as the observed inflammatory responses may partially reflect surgical stress rather than differences between single and multiple joint replacements. Future studies could address this by including baseline inflammatory data or control groups to better differentiate the effects of surgical stress from systemic inflammatory responses. Using specific biomarkers that distinguish between systemic and localized inflammation would also enhance the precision of future analyses.

In addition, the considerable heterogeneity within our study population may have impacted the internal validity of our findings and limited the generalizability of our results. This heterogeneity increases the risk of confounding bias and selection bias, potentially influencing the robustness of our conclusions. To address these limitations, future research should include larger sample sizes and employ stratified analyses and multivariate adjustments to better account for population variability.

Finally, this study focused exclusively on the immediate postoperative period (1–14 days), leaving long‐term outcomes, including functional recovery, quality of life, and late‐onset complications, unexplored. This limitation prevents a comprehensive evaluation of the durability, safety, and efficacy of single versus multiple joint replacements over time.

To address these limitations, future research should consider larger, multicenter cohorts to enhance statistical power and generalizability. Prospective study designs with standardized protocols for data collection would minimize bias and allow for better control of confounding variables. Comprehensive documentation of perioperative management strategies and the inclusion of long‐term follow‐up data will be essential for assessing sustained outcomes and late complications. Additionally, randomized controlled trials are needed to validate these findings, mitigate selection bias, and provide high‐quality evidence to guide clinical decision‐making for hemophilic patients undergoing joint replacement surgery.

## 5 Conclusion

This study provides valuable insights into the perioperative management of patients with hemophilic arthritis undergoing single versus MJR surgeries. The findings reveal no significant differences in the risk of infection, bleeding, or postoperative transfusion requirements between single and multiple joint replacements. Importantly, patients undergoing multiple joint replacements demonstrated superior postoperative joint mobility, suggesting that multi‐joint replacement surgery may offer functional advantages without increasing the risk of complications.

These results have significant implications for clinical practice. First, we found that single (SJR) and multiple joint replacements (MJR) have similar risks of infection and bleeding in hemophilia patients when managed properly. This shows that MJR can be safely done. Second, since SJR and MJR have similar inflammation responses, planning should focus on each patient's needs and goals, not just the size of the surgery. Lastly, close monitoring and personalized care during and after surgery are crucial for good results.

Future research should look at how SJR and MJR affect patients' daily life and well‐being in the long term. We also need to improve surgical care plans based on our findings to make joint replacements safer and more effective. This will help doctors make better decisions and improve care for people with hemophilia.

## Author Contributions

L.S. was involved in conception and design, and data interpretation for this article. M.S. was involved in manuscript drafting. M.S., P.Q., W.J., S.Y., G.C., Z.Z., X.W. were involved in data collection, case diagnosis and confirmation for this article. J.W., X.N. were involved in data analysis for this article. L.S. was involved critical review in for this article. All authors reviewed the manuscript.

## Ethics Statement

The study complied with the Declaration of Helsinki and was approved by the Ethics Committee of Shenzhen Third People's Hospital (Contract number: 2022–119).

## Consent

Written informed consent was obtained from all participants.

## Conflicts of Interest

The authors declare no conflicts of interest.
